# A Narrative Literature Review of the Established Safety of Human Serum Albumin Use as a Stabilizer in Aesthetic Botulinum Toxin Formulations Compared to Alternatives

**DOI:** 10.3390/toxins15100619

**Published:** 2023-10-18

**Authors:** Sonja Sattler, Stephen Gollomp, Andrew Curry

**Affiliations:** 1 Rosenpark Klinik GmbH, 64297 Darmstadt, Germany; sonja.sattler@rosenparkklinik.de; 2 Lankenau Medical Center, Wynnewood, PA 19096, USA; gollomps@mlhs.org; 3 Merz Aesthetics, Raleigh, NC 27615, USA

**Keywords:** human serum albumin, polysorbates, adverse events

## Abstract

Despite more than 80 years of use in a number of conditions, including in critically ill patients, comments have recently arisen regarding the safety and efficacy of human serum albumin (HSA) as a therapeutic product and stabilizer/excipient in botulinum neurotoxins. This review summarizes the literature on the safety of HSA. Beyond decades of safe use, the largest clinical dataset of HSA safety is a large meta-analysis of HSA supplier data, which found only an extremely remote risk of serious adverse events across millions of doses of therapeutic concentrations of HSA. There is a paucity of literature identifying HSA-specific adverse events when used as a stabilizer/excipient; however, studies of HSA-containing botulinum neurotoxins (BoNTs) suggest that adverse events are not related to HSA. Polysorbates, which are synthetically produced and not physiologically inert, are contained in pending or new-to-market BoNT formulations. In contrast to HSA, evidence exists to suggest that polysorbates (particularly PS20/PS80) can cause serious adverse events (e.g., hypersensitivity, anaphylaxis, and immunogenicity).

## 1. Introduction

Human serum albumin (HSA), with a blood concentration of 35–50 mg/mL, is the most abundant protein in plasma and is integral in maintaining osmotic blood pressure [1] and transporting diverse molecules, such as water, metabolites, gas, and exogenous substances, throughout the body [2]. HSA contains 17 disulfide bonds, which not only provide stability and resilience to environmental stress, but also provide binding sites, which give it the ability to interact with a range of pharmaceutical products to stabilize them in various situations [3]. HSA is easily broken down into amino acids and peptides, which are reabsorbed into the intestinal tract [1]. Prepared from pooled human plasma and not containing isoagglutinin or blood group substances, HSA can be administered, independent of blood type [2]. Based on manufacturing and pathogen inactivation processes, albumin preparations are considered to carry no risk of transmitting infections [2]. The physical properties of HSA make it an extremely versatile and multifunctional protein, which is why it is so widely used by the pharmaceutical industry; HSA’s pharmacokinetic properties are utilized in drug-delivery systems, while its ability to increase the solution stability of other proteins is used in pharmaceutical formulations [4].

Clinical administration of HSA began in the early 1940s, when it was first used to treat severely burned victims of the attack on Pearl Harbor [1]. Today, HSA is widely used to treat several conditions and diseases, including life-threatening conditions such as surgical blood loss, trauma, shock, burns, hemorrhage, acute respiratory distress syndrome, hemodialysis, acute liver failure, and hypoalbuminemia, as well as for cardiopulmonary bypass, nutrition support, and resuscitation [1]. HSA is also frequently used as an acute volume replacement in the intensive care unit (ICU), as well as in pregnant and neonatal patients [2,5].

To remain biologically active, proteins contained within pharmaceutical products must maintain a stable, three-dimensional structure during production, storage, and transportation, as there is potential for exposure to a variety of stresses such as temperature variations and pH changes [6]. While oxidation is a common cause of degradation, particularly during storage, in both liquid and solid formulations of pharmaceutical products, loss of therapeutic proteins through nonspecific adsorption can also lead to structural changes, denaturation, and inactivation [7]. Additionally, aggregation during production and/or delivery is a concern, primarily due to the increased potential for aggregates to lead to an immunogenic reaction and to complications during administration of products [8]. To maintain stability of pharmaceutical products, surface-active agents, or surfactants, are frequently added [6]. Because HSA is able to provide protection from aggregation, non-specific adsorption, and oxidation, as well as improve solubility and consistency [9,10,11,12], it is often used as this cell-culture-medium supplement (excipient) in the production of vaccines, pharmaceutical drugs, and biologics [1].

Despite the long history of safety with HSA, polysorbates (e.g., PS20 and PS80) have been recently touted as potentially better stabilizers without the safety and supply concerns HSA [13]. Currently, polysorbates are used in multiple biopharmaceutical drugs according to the Physicians’ Desk Reference; the concentrations in formulations vary, ranging from 0.0003% to 0.3% weight by volume [6,14]. While polysorbates are used as stabilizers against surface adsorption, under certain conditions product degradation can result in protein modification and degradation with unknown consequences [6]. Additionally, commercially available polysorbates are chemically diverse mixtures with heterogeneity arising from the hydrophilic head group and fatty acid tail, as well as from process-related impurities, levels of byproducts, and the raw materials used [15].

HSA may be one of the most universal therapeutics in the biomedical field, with a safety record that has been long-established across a wide range of applications in pharmaceuticals. This review summarizes the literature, supporting the safety of HSA as a stabilizer/excipient and highlighting its safety record in comparison to its suggested synthetic alternative, polysorbate.

## 2. Summary of Human Serum Albumin Safety

### 2.1. Adverse Events

There are limited data focusing on the safety and tolerability of HSA, specifically as a stabilizer/excipient, despite the fact that its ability to stabilize protein-based pharmaceuticals at low concentrations was established decades ago [4]. Using data from 10 major therapeutic suppliers of HSA worldwide, a meta-analysis found that the observed incidence of adverse events (AEs) of HSA between 1998–2000 indicated that non-fatal and fatal serious adverse events (SAEs) are exceedingly rare [16], confirming results of an earlier meta-analysis of supplier data for 1990–1997 [17]. Within the supplier data, SAEs were defined as AEs involving death and life-threatening clinical conditions, hospitalization or extended hospitalization, disability, congenital anomaly, or intervention to prevent permanent impairment or damage. Of approximately 16.2 million doses (1.62 × 10^7^) of 40 g of HSA administered between 1998 and 2000, there were 198 non-fatal SAEs and 13 fatal SAEs [16]. Per million doses, the incidence (95% confidence interval) of non-fatal and fatal SAEs was 4.65 (1.34, 16.2) and 0.770 (0.345, 1.72), respectively. Of the 198 non-fatal SAEs, 76 (38.4%) were considered possibly related to HSA and 7.1% were considered probably related to HSA, corresponding to incidences (95% confidence interval) of 1.14 (0.278, 4.66) and 0.208 (0.0186, 2.33), respectively, per million doses. The nature of non-fatal SAEs was not reported. Of the 13 fatalities, 3 were considered possibly related to HSA (anaphylactic shock, toxicoderma and respiratory insufficiency, and cardiac arrest and apnea), and none were considered probably related [16]. Additionally, it was determined that the increase in incidence between 1990–1997 and 1998–2000 for non-fatal and fatal SAEs with HSA administration was primarily attributed to SAEs not directly related to HSA.

### 2.2. Human Serum Albumin Use in Intensive Care Units

Beginning in the 1950s, albumin has been utilized to reduce morbidity and mortality in preterm neonates, initially in respiratory distress syndrome [18]. Since then, HSA infusions have been commonly used in the neonatal ICU for volume expansion, inflammatory disorders, increased capillary permeability, hypoalbuminemia, indirect hyperbilirubinemia, and protein-losing conditions [19]. Fluid resuscitation constitutes a mainstay in the management of patients in the ICU, with crystalloids, non-protein colloids, and HSA being widely utilized [20]. In the general ICU population, HSA has shown to be a safe intravenous fluid [21], and may also be useful in “small volume resuscitation” (i.e., achievement of same hemodynamic endpoints with significantly smaller volumes administered), although further large-scale research is needed [22]. Furthermore, HSA is used as a formulation buffer for intravenous infusion in two antibody products for the management of cancer [23].

Hypoalbuminemia (serum albumin < 35 g/L) is commonly seen in ICU settings in up to 76% of critically ill children [24] and up to 82% in certain critically ill adults [25]. A meta-analysis of 90 studies, including more than 290,000 patients, found that, for each 10-g/L decline in HSA, there was a 137% increase in odds of mortality and a 89% increase in odds of morbidity (i.e., complications) [26]. The meta-analysis also found a 26% decrease in odds of morbidity with HSA administration; however, the results were not significant [26]. A single-center, open-label pilot study of 100 patients with HSA levels ≤30 g/dL found that daily administration of HSA, compared to no HSA administration, significantly improved organ function and resulted in less fluid overload [27]. A subsequent randomized controlled trial could not replicate these results, showing no differences in survival or sustained improvement in organ dysfunction, possibly due to use of greater concentrations of HSA (20% vs. 4%), which resulted in the administration of markedly lower volumes than in the previous study [28]. Albumin is a well-established therapy in patients with liver disease (e.g., advanced liver cirrhosis), reducing the risk of death up to 38% compared to those who received standard medical therapy [29]. Although consensus opinion favors the use of albumin in large paracentesis and in spontaneous bacterial peritonitis, the use of albumin for other acute hepatic syndromes is less clear [30].

### 2.3. Immunogenicity

HSA allotypes, or variant albumin identified in the population, may have different immunogenic properties than normal HSA or albumin A [31]. Overall, no evidence of immunogenicity with HSA administration has been found. One study analyzing blood samples from a subset of 39 patients found no instance of specific antibodies developed against HSA when used as a stabilizer in allergy extracts [32]. In addition, no substance-specific, clinically relevant alterations in organ function due to HSA therapy have been reported [2]. Although HSA is prepared from pooled plasma from many donors, HSA preparations are considered non-immunogenic [2]. In rare cases, slight reactions such as flush, urticaria, elevated temperature, and nausea developed, but were resolved quickly [2].

### 2.4. Infectivity/Pathogen Transmission Risks

Studies examining infectivity and virus inactivation in HSA products have focused on large doses of albumin used for infusions (typically 20 g/dose) [33]. A systematic review found five studies that examined the rate of transmission of hepatitis B virus from HSA products after being heat-treated/pasteurized with no evidence of viral infection, even when the plasma contained high levels of infective hepatitis B virus [33].

There is no evidence suggesting transmission of prion-type agents to humans through any blood products causing Creutzfeldt-Jakob disease (CJD) or other prion diseases. Additional precautions, such as exclusion of potential donors who report family members with CJD, or treatments associated with CJD, have been taken by blood collection organizations [33]. Epidemiological studies reviewed by the United States Food and Drug Administration (US FDA) guidance found no evidence linking CJD to the receipt of blood or blood products, and only limited evidence linking variant CJD to plasma transfusions exists (four patients in the United Kingdom) [34]. Currently, plasma derivatives have not been implicated in CJD transmission in any country other than the United Kingdom, and based on the US FDA guidelines, only carry a theoretical risk for transmission of CJD. 

## 3. Current Use of Human Serum Albumin in Botulinum Toxin Products

HSA as a stabilizer/excipient is found in the majority of botulinum neurotoxins (BoNTs) for therapeutic and cosmetic use, including onabotulinumtoxinA (Botox^®^), incobotulinumtoxinA (Xeomin^®^), abobotulinumtoxinA (Dysport^®^), and prabotulinumtoxinA (Jeuveau^®^) (Table 1).

BoNTs are synthesized as a progenitor toxin complex by *Clostridium botulinum* and related species [45,46], to which HSA is added to stabilize the neurotoxin. An ex vivo mouse phrenic nerve hemidiaphragm assay found that increasing the concentration of HSA resulted in a magnification of the paralytic effect of BoNT type A [46]. This dose-dependent effect was observed up to concentrations of 0.8 mg/mL, after which the effect plateaued, leading authors to suggest that maximal protection of BoNT type A occurs at 0.8 mg/mL of HSA [46]. HSA concentrations in BoNT type A formulations currently on the market range from 0.125 to 1.0 mg per vial, and are significantly lower than those utilized in the cases of intensive care or transfusions, which are deemed safe, with little to no AEs.

Whether adverse reactions to treatment with BoNTs containing HSA are associated specifically with HSA is typically not determined; however, the nature of the AEs would suggest that they are unrelated to treatment (e.g., worsening of disease-related symptoms), related to the active ingredient (e.g., muscular weakness), or related to administration (e.g., injection-site reactions [ISRs]). Frequently reported adverse reactions with BoNTs containing HSA for cosmetic indications are summarized in Table 2.

## 4. Summary of Polysorbate Safety

Polysorbate 20 and 80 (PS20 and PS80) are used as surfactants, stabilizers, and emulsifiers in cosmetics, industrial detergents, foods, and drugs [15]. Most commercial therapeutic protein formulations use PS20 and PS80 as protein stabilizers due to biocompatibility, low toxicity, high hydrophile–lipophile balance value, and low critical micelle concentrations (Table 1) [15]. Polysorbates belong to the family of ethoxylated non-ionic surfactants and are polyoxyethylene-1,4-sorbitan-monoesters of fatty acids; the current understanding of mechanisms of action do not suggest any differentiation between PS20 and PS80 [47]. Despite the US FDA granting PS80 a “generally recognized as safe” status, and both PS20 and PS80 being listed in its Inactive Ingredients Database [48], it is widely recognized that polysorbates are associated with injection- and infusion-site adverse events, hepatotoxicity, pseudoallergy, hypersensitivity reactions, and anaphylactic shock [49,50,51,52]. 

### 4.1. Adverse Events

Polysorbates are prone to degradation by oxidation and hydrolysis, and may inadvertently impact product quality, efficacy, safety, and stability. PS80 degrades via hydrolysis, which causes a slower surface adsorption rate, and free fatty acid release from hydrolysis forms insoluble particles, negatively impacting protein quality and stability [6,15]. As a result of degradation, the oxidation of monoclonal antibodies may occur, thus increasing immunogenicity; reactive products may also develop and interact with proteins at the injection site leading to ISRs [53]. Auto-oxidation of PS80 is the most common degradation route and can be caused by opening a packing bottle without nitrogen overlay, trace metal contaminants, and presence of iron [54]. Polysorbates are not physiologically inert and systemic administration of PS80 at high doses (300 mg in 10 min) can lead to transient hypotension and tachycardia in patients [47]. 

#### Hypersensitivity/Injection-Site Reactions

Even as inactive ingredients, polysorbates can cause hypersensitivity, ISRs, contact dermatitis, urticaria/angioedema, and anaphylaxis through activation of the complement system [53]. Both PS20 and PS80 can directly activate complement, leading to a significant production of pleiotropic proinflammatory mediator C3a in human blood [47].

Based on two cases with prior history of immediate hypersensitivity to polyethylene glycol (PEG)-containing medications, skin testing and oral challenges with PEG and polysorbate-containing agents determined clinical reactivity and cross reactivity between the two allergens [55]. Cross-reactive immediate hypersensitivity to polyether-containing compounds, such as polysorbates, can occur via Type I hypersensitivity mechanisms [55]. Based on a literature-based investigation, examining reasons for the development of ISRs during monoclonal antibody therapies, polysorbate-induced hypersensitivity may contribute to ISRs and some SAEs in biologics [47]. Systemic administration of IV docetaxel (Docefrez^®^) and cabazitaxel (Jevtana^®^)—both of which have high PS80 concentrations—led to hypersensitivity reactions in 5–40% of treated patients, despite pretreatment with antihistamines and corticosteroids [56,57]. Additionally, hypersensitivity in patients who received darbepoetin and erythropoietin was due to the PS80 product [50,58]. Other patients have developed generalized urticaria, angioedema, rhinoconjunctivitis, dyspnea, and wheezing 1 h after the third intramuscular administration of the human papilloma virus vaccine, which has been determined to be due to PS80 [59]. Of the patients treated with omalizumab (Xolair^®^), 45% had reports of ISRs, which were determined to be related to PS20 [47].

Polysorbates may elicit type IV immunological reactivity; free radical species and other oxidation products of polysorbates (e.g., aldehydes, ketones) function as haptens and may react with proteins at exposure site to form neoantigens, resulting in the subsequent activation of the immune system [47]. Such immunological responses were evaluated in the recently developed COVID-19 vaccines, which contain PEG and polysorbates as excipients.

### 4.2. Botulinum Toxin Products Containing Polysorbates 

Whereas all other BoNT products rely on HSA as an excipient to limit aggregation and adsorption of BoNT molecules to glass surfaces, newly developed BoNT formulations (relabotulinumtoxinA, liquid botulinum toxin type A, BoNT type A MT10107, and daxibotulinumtoxinA) have shifted to polysorbate as an excipient (Table 1). While daxibotulinumtoxinA (Daxxify^®^)—a product using a virally derived excipient peptide (RTP004) in addition to PS20—has a documented treatment-related AE rate of 20% [44], a higher incidence of AEs was seen in trials for liquid BoNT type A (Alluzience^®^) (Table 2) [42]. As these PS20/80-containing BoNT formulations are either pending or very new to the market, long-term safety is unknown.

## 5. Discussion

This review addressed a literature gap on the safety of HSA as a stabilizer/excipient, particularly in aesthetic injectables. The literature demonstrates a long-established pattern of enviable safety records throughout multiple use applications of HSA in clinical practice and in pharmaceutical products. Notably, much of the safety of HSA has been established in larger volume administration, leaving little concern for its use in aesthetic injectables, in which the amount of HSA is substantially less. Conversely, polysorbates, a synthetic ingredient, demonstrate a mixed or unknown safety record in drug or biologic products.

As the most abundant protein in plasma, HSA has a long history of safe use in many treatment contexts (e.g., for blood volume replacement in the ICU and as an excipient in vaccines and aesthetic injections) with very little evidence of immunogenicity or transmission of any blood-borne pathogens, even at large amounts (Table 3). The physical properties responsible for the biological function of HSA also make it the perfect stabilizer for many pharmaceutical products, especially products in liquid formulations, which can be vulnerable to surface adsorption during the manufacturing and/or storage processes to silicone tubing, glass or plastic containers, or drug delivery devices. Reports of AEs, particularly in large amounts, appear to be minimal, and because of its abundance and therefore natural tolerance in the human body, the likelihood of HSA eliciting an immune response in a given patient population is minimal.

AEs reported for BoNTs utilizing HSA have not been considered related to HSA and are more likely related to the administration or the formulation of the toxin itself, as the biggest structural difference between BoNT formulations is the presence or absence of complexing proteins, the presence of which has been reported to increase the risk of neutralizing antibody formation [60]. Use of polysorbates (particularly PS20/PS80) in drug products has led to hypersensitivity, ISRs, contact dermatitis, anaphylaxis, and immunogenicity in patients, regardless of volume. The quality of polysorbate solutions may have increased, now including highly purified, low-peroxide, low-acid-content solutions [6]; however, degradation may still occur, and the lowest amount of polysorbate possible should be used to minimize risk of damage to protein products. Many reported cases of clinical adverse effects may be due to degradation of polysorbates, which negatively impacts the product and its stability, deeming it ineffective and unsafe [47]. Additionally, the commercially available “new” or novel excipients, such as PS20/PS80, should undergo safety assessments prior to use; however, existing excipients are generally only evaluated indirectly via the overall safety profile of the entire product [47]. 

## 6. Conclusions

The long-established safety record of HSA across a broad range of pharmaceutical applications is supported by a large amount of evidence indicating minimal adverse reactions of HSA, even when administered in large volumes. Moreover, when used as a stabilizer/excipient in injectables, the amount is magnitudes less than when used as a therapeutic agent, minimizing the already minimal chance of adverse reactions even further. As a naturally occurring protein in human blood, HSA possesses properties like biocompatibility, biodegradability, non-immunogenicity, and nontoxicity, making it a safe stabilizer/excipient option in aesthetic injectables.

## Figures and Tables

**Table 1:** Botulinum toxin excipient usage and approval.

Product (US Brand Name)	Excipient Used (Dosage per 100 U Vial) ^1^	Approval ^2^ (Year)
OnabotulinumtoxinA (Botox^®^) [35]	HSA (0.5 mg)	US FDA (1989); 98 other countries
IncobotulinumtoxinA (Xeomin^®^) [36]	HSA (1 mg)	EMA (2009); US FDA (2010)
AbobotulinumtoxinA (Dysport^®^) [37]	HSA (0.125 mg/300 U)	US FDA (2009); 75 other countries
PrabotulinumtoxinA (Jeuveau^®^) [38]	HSA (0.5 mg)	US FDA (2019)
LetibotulinumtoxinA [39]	HSA (0.5 mg)	Under US FDA review; 44 other countries
RelabotulinumtoxinA [QM114] [40,41]	Polysorbate 80 (*)	Investigational
Liquid botulinum toxin type A (Alluzience^®^) [42]	Polysorbate 80 (*)	EMA (2021)
Botulinum toxin type A [MT10107] (Coretox) [43]	Polysorbate 20 (0.02 mg/kg)	South Korea (2022)
DaxibotulinumtoxinA (Daxxify^®^) [44]	Polysorbate 20 (0.1 mg)	US FDA (2022)

^1^ Dosage forms used in cosmetic indications; per 100 U vial unless otherwise stated. ^2^ First approval for any indication. * Not publicly disclosed. EMA, European Medicines Agency; HSA, human serum albumin; U, units; US FDA, United States Food and Drug Administration approval for any indication.

**Table 2:** Most frequently reported adverse reactions in botulinum toxins containing human serum albumin and polysorbate.

Product (US Brand Name)	Cosmetic Indications	Adverse Reactions Reported in ≥1% of Patients
BoNTs containing HSA		
OnabotulinumtoxinA (Botox^®^) [35]	Glabellar lines Lateral canthal lines Forehead lines	(3%) eyelid ptosis (1%) eyelid edema (9%) headache and (2%) brow ptosis
IncobotulinumtoxinA (Xeomin^®^) [36]	Glabellar lines	(>1%) headache
AbobotulinumtoxinA (Dysport^®^) [37]	Glabellar lines	(≥2%): nasopharyngitis, headache, injection-site pain, ISR, upper respiratory tract infection, eyelid edema, eyelid ptosis, sinusitis, nausea, and blood present in urine
PrabotulinumtoxinA (Jeuveau^®^) [38]	Glabellar lines	(≥1%): headache, eyelid ptosis, upper respiratory tract infection, and increased white blood cell count
LetibotulinumtoxinA [39]	Glabellar lines	(≥3%): eyelid ptosis and ISR
BoNTs containing polysorbate		
RelabotulinumtoxinA [QM114] [40,41]	Glabellar lines Lateral canthal lines	(3.6%) headache and (6.1%) injection site bruising
Liquid botulinum toxin type A (Alluzience^®^) [42]	Glabellar lines	(≥10%): headache, ISRs, asthenia, fatigue, and influenza-like illness
Botulinum toxin type A (MT10107; Coretox) [43]	Glabellar lines	−−
DaxibotulinumtoxinA (Daxxify^®^) [44]	Glabellar lines	(≥1%): headache, eyelid ptosis, and facial paresis (including facial asymmetry)

BoNT, botulinum neurotoxin; HSA, human serum albumin; ISR, injection-site reaction; US, United States.

**Table 3:** Comparison of human serum albumin and polysorbate 20/80 use.

	Human Serum Albumin	Polysorbate 20/80
Primary use in medicine	Cell culture medium, excipient/protein stabilizer in vaccines/pharmaceuticals, acute volume replacement in ICU setting	Excipient/protein stabilizer in vaccines/pharmaceuticals, industrial detergents, foods
Documented adverse events	In rare cases, reactions such as flush, urticaria, elevated temperature, and nausea can occur following administration	Transient hypotension, tachycardia, hypersensitivity, ISRs, anaphylaxis, instability of protein product due to polysorbate hydrolysis
Immunogenicity	No instances of specific antibodies developed against human serum albumin	Hypersensitivity via complement activation, degradation results in oxidation products and free radicals which function as haptens activating immune system, anaphylaxis
Infectivity/pathogen transmission risk	Theoretical risk of hepatitis B virus and CJD transmission, but low to absent evidence	NR

CJD, Creutzfeldt-Jakob disease; ICU, intensive care unit; ISR, injection-site reaction; NR, not reported.

## Data Availability

Not applicable.
